# Associations of dietary inflammation index and composite dietary antioxidant index with all-cause mortality in COPD patients

**DOI:** 10.3389/fnut.2025.1514430

**Published:** 2025-01-21

**Authors:** Sue Zhao, Yingjie Su, Hongzhong Yang

**Affiliations:** ^1^Department of Pulmonary and Critical Care Medicine, Changsha Central Hospital, Changsha, China; ^2^Changsha Central Hospital, Changsha, China

**Keywords:** chronic obstructive pulmonary disease, dietary inflammatory index, composite dietary antioxidant index, mortality, NHANES

## Abstract

**Background:**

Few studies have investigated the effects of both dietary inflammatory index (DII) and composite dietary antioxidant index (CDAI) on mortality in patients with Chronic Obstructive Pulmonary Disease (COPD). Our research aimed to explore the associations between the two indicators with all-cause mortality in COPD patients.

**Methods:**

We conducted a prospective cohort analysis based on data from the six cycles of the National Health and Nutrition Examination Survey (NHANES) dataset from 2007 to 2018. Multivariate Cox proportional hazard models were used to analyze the effects of DII and CDAI on all-cause mortality in COPD. We employed restricted cubic spline (RCS) analysis to examine the dose–response relationship between two indicators and all-cause mortality, used threshold effect analysis to determine the inflection point, and conducted subgroup analysis and interaction tests to verify the stability of the results.

**Results:**

A total of 1,457 COPD patients aged over 40 were enrolled in the study. The median follow-up time was 76.8 months. The multivariate Cox proportional hazards model showed that increased DII was associated with an increase in all-cause mortality (HR (95% CI): 1.11(1.04, 1.18), *p* = 0.002). In contrast, CDAI was negatively correlated with all-cause mortality (HR (95% CI): 0.95(0.91, 0.99), *p* = 0.01). The RCS analysis showed a nonlinear correlation between DII or CDAI and all-cause mortality. The maximum pro-inflammatory inflection point of DII was 2.32, while the antioxidant threshold of CDAI is −0.12. Subgroup analyses indicated that the relationship between exposure variables and all-cause mortality was stable in most populations.

**Conclusion:**

Reducing the pro-inflammatory diet or increasing the antioxidant diet can reduce all-cause mortality in COPD patients.

## Introduction

Chronic obstructive pulmonary disease (COPD) is a heterogeneous disease characterized by chronic respiratory symptoms (dyspnea, cough, phlegm, acute exacerbation), which are caused by airway (bronchitis, bronchiolitis) and/or alveolar abnormalities (emphysema), causing continuous progressive exacerbation of restricted airflow (1). The pathogenesis of COPD primarily involves chronic airway inflammation, and oxidative and antioxidant imbalance. It is estimated that there are 300 to 400 million patients with COPD worldwide (2), making it the third leading cause of death globally (3), with approximately 3 million people dying from COPD each year. By 2050, the number of COPD patients is projected to reach 600 million (4). Given the significant health risks associated with COPD, exploring potential strategies to reduce mortality is essential to extend patients’ lives and alleviate the global burden of disease.

Dietary patterns have been proven to be related to many diseases. Current studies indicate that inflammatory diets increase all cause mortality including the risk of death from cancer and cardiovascular diseases (CVD) (5). On the other hand, diets with higher antioxidant capacity are associated with lower all-cause, cardiovascular, and respiratory disease mortality (6, 7).

The dietary inflammation index (DII) is an indicator based on the population’s intake of dietary nutrients. It is used to assess the inflammatory potential of diets (8), and is most widely used in epidemiological studies in order to explore the association between dietary inflammation potential and diseases (9–12). The composite dietary antioxidant index (CDAI) was developed to assess the antioxidant capacity of dietary intake (13).

Many studies have indicated that dietary factors are related to the onset and progression of COPD (14, 15). Two prospective cohort studies showed a positive relationship between DII and all-cause mortality in COPD patients or those with sarcopenia in the United States (16, 17). Another cross-sectional study found that CDAI is linearly negatively correlated with COPD risk (18). Moreover, cross-sectional and prospective cohort studies have confirmed that co-exposure to DII and CDAI has an impact on lung function [forced expiratory volume in 1 s (FEV1), forced vital capacity (FVC), preserved ratio impair spirometry (PRISm)] and all-cause death in patients with asthma (19, 20). At present, there has been no research conducted on the impact of both a pro-inflammatory diet and an antioxidant diet on COPD mortality. We aim to determine the effect of co-exposure to DII and CDAI on all-cause mortality in COPD patients aged 40 and above by utilizing the data from the National Health and Nutrition Examination Survey (NHANES) database from 2007 to 2018.

## Methods

### Study design and population

The NHANES is conducted by the Centers for Disease Control and Prevention (CDC) in the United States, which evaluates the nutritional and health status of the U.S. population through interviews, collection of demographic and dietary data, laboratory tests, and physical measurements. NHANES is a free and open database that has been approved by the Ethics Review Committee of the National Center for Health Statistics. All participants signed written informed consent.

**Figure 1:**
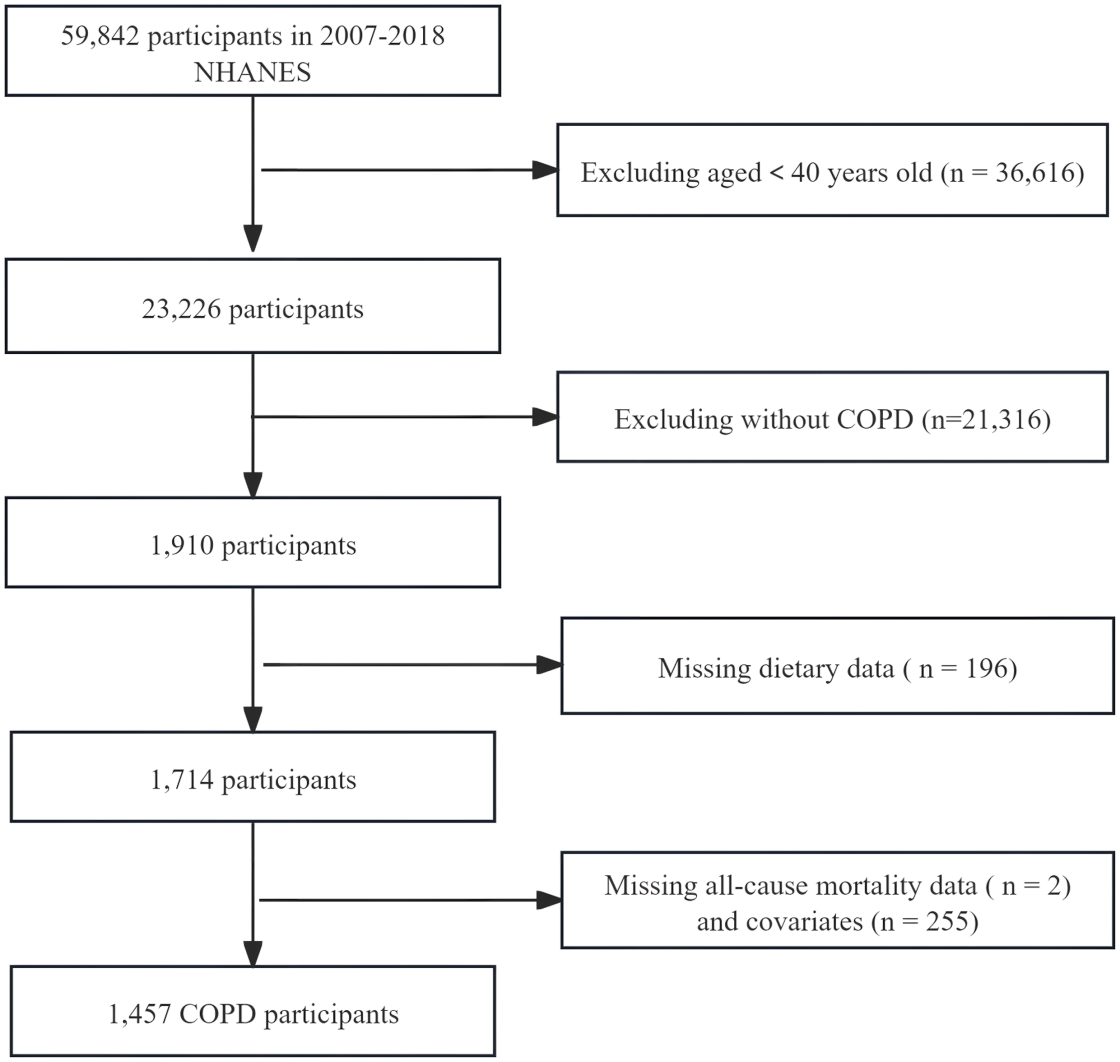
Flowchart of the standard for participants enrolled in the study.

We selected information from six cycles of the NHANES database from 2007 to 2018 for analysis. The diagnosis of COPD was met by fulfilling any of the following conditions: (1) Having been informed by a doctor of COPD or emphysema; (2) FEV1/FVC < 0.7 post bronchodilator; (3) Age 40 or over, with a history of chronic bronchitis or smoking, and using the following medications: inhaled corticosteroids, selective phosphodiesterase-4 inhibitors, leukotriene modifiers, mast cell stabilizers (16). Exclusion criteria: Individuals under 40 years of age, those lacking dietary data or follow-up information, and individuals with missing covariates. We ultimately included data from 1,457 COPD participants in this analysis (Figure 1).

### Evaluation of DII

The dietary data used for calculating the DII score was collected through 24-h dietary recall interviews or food diaries. The DII, based on 45 specific nutrients, was used to assess the impact of dietary consumption on inflammation. Our study utilized 28 nutrients to calculate the DII score, excluding supplements or medications, encompassing energy, cholesterol, total fat, monounsaturated fatty acids, n-3 fatty acids, n-6 fatty acids, polyunsaturated fatty acids, saturated fat, proteins, carbohydrates, fiber, alcohol, vitamins A/C/D/E/B12/B6, beta-carotene, caffeine, folic acid, magnesium, iron, niacin, riboflavin, thiamin, selenium, and zinc. The formula for calculating DII was as follows (8). The higher the score, the stronger the pro-inflammatory effect of the diet.


$$\begin{array}{l} Z\;\mathrm{score}=\left(\mathrm{daily}\;\mathrm{mean}\;\mathrm{intake}–\mathrm{global}\;\mathrm{daily}\;\mathrm{mean}\;\mathrm{intake}\right)\\ /\mathrm{standard}\;\mathrm{deviation} \end{array}$$

$$Z\;{\mathrm{score}}^{’}=Z\;\mathrm{score}\to \left(\mathrm{converted to}\;a\;\mathrm{percentile score}\right)\times 2–1$$

$$DII=\left(Z\;{\mathrm{score}}^{’}\times \mathrm{overall inflammatory effect score}\right)$$

### Evaluation and calculation of CDAI

Wright et al. developed the CDAI calculation method (13). Based on a 24-h dietary review, the intake of six antioxidants vitamin A, vitamin C, vitamin E, carotenoids, zinc, and selenium was calculated. The calculation formula was as follows (19):


$$\begin{array}{l} \mathrm{CDAI}=\left(\mathrm{daily}\;\mathrm{mean}\;\mathrm{intake}–\mathrm{global}\;\mathrm{daily}\;\mathrm{mean}\;\mathrm{intake}\right)\\ /\mathrm{standard}\;\mathrm{deviation} \end{array}$$

### Ascertainment of deaths

All-cause mortality data was associated with the NHANES National Mortality Index, followed up to December 31, 2019.

### Covariates

Based on previously published literature (19), potential confounders that may affect dietary quality and mortality were included. Alcohol consumption and energy intake were included in the DII, so they were not included. The confounders included age, gender, race, education, poverty-income ratio (PIR) (<1, 1–3, ≥3) (21), body mass index (BMI), smoking history (smoking ≥100 cigarettes in life or not), alanine transaminase (ALT), aspartate transaminase (AST), white blood cells (WBC), creatinine, urea nitrogen, and physical activity. It also included a history of comorbid diseases: hypertension, diabetes, CVD, and cancer. Detailed measurement procedures can be found on the NHANES official website.

### Statistical analysis

Given the multi-stage sample survey design adopted by NHANES, we used appropriate sampling weights (wtmec2yr) for analysis. Categorical variables are displayed as counts (weighted percentages), and continuous variables are represented by weighted means (standard errors). We conduct a weighted univariate Cox regression analysis to examine the relationship between the variables and all-cause mortality. DII and CDAI were converted to categorical variables based on tertiles. We used multivariate Cox regression models to study the link between DII and CDAI with all-cause mortality of COPD. Three models were established. Model 1, covariates were not adjusted; Model 2, adjusting for age, gender, and race; Model 3, adjusting for age, gender, race, education level, PIR, BMI, smoking history, WBC, AST, creatinine, urea nitrogen, exercise, hypertension, diabetes, CVD, and cancer history. Furthermore, we employed the Restricted Cubic Spline (RCS) method to analyze the dose–response relationship between exposure variables and all-cause mortality. If the relationship was non-linear, we further analyzed the connection between DII or CDAI and all-cause mortality on both sides of the inflection point using a two-piece Cox hazard ratio (HR).

In addition, subgroup analysis and interaction tests were performed to verify whether the influence of DII or CDAI on all-cause death in the entire cohort was stable across subgroups. All statistical analyses were carried out using the R software package (version 4.2.2) and Empower Stats (X&Y Solutions Inc). *p* < 0.05 was defined as statistically significant.

## Results

1. Eventually, a total of 1,457 patients with COPD were included. Males accounted for 51.58% of all the citizen representatives. Table 1 described the clinical characteristics of the participants, categorized by survival outcomes. Deceased patients had higher DII scores and lower CDAI scores.
2. Univariate weighted Cox regression analysis showed that both DII and CDAI were associated with all-cause mortality in COPD (Table 2).

**Table 1:** Features of the study population, weighted.

Variables	Total (*n* = 1,457)	Alive (*n* = 1,068)	Deceased (*n* = 389)	*p*- value
Age (years), *n* (%)				<0.0001
<60	499 (41.50)	440 (47.61)	59 (17.78)	
≥60	958 (58.50)	628 (52.39)	330 (82.22)	
Gender, *n* (%)				0.98
Female	645 (48.42)	498 (48.44)	147 (48.34)	
Male	812 (51.58)	570 (51.56)	242 (51.66)	
Race, *n* (%)				0.02
Non-Hispanic White	961 (82.82)	676 (81.74)	285 (87.01)	
Non-Hispanic Black	240 (6.69)	180 (6.61)	60 (6.98)	
Mexican American	69 (1.57)	59 (1.74)	10 (0.90)	
Other Hispanic	94 (2.38)	77 (2.60)	17 (1.53)	
Other Race	93 (6.54)	76 (7.31)	17 (3.58)	
Education level, *n* (%)				0.001
Less than high school	429 (21.10)	292 (18.88)	137 (29.72)	
High School or equivalent	386 (27.72)	284 (28.39)	102 (25.12)	
College Graduate or above	642 (51.18)	492 (52.73)	150 (45.15)	
PIR, *n* (%)				<0.0001
<1	345 (16.86)	250 (15.72)	95 (21.26)	
1–3	695 (40.71)	482 (38.15)	213 (50.64)	
≥3	417 (42.44)	336 (46.13)	81 (28.09)	
BMI (kg/m2), *n* (%)				0.24
<25	384 (25.48)	258 (24.44)	126 (29.49)	
25–30	465 (32.54)	345 (33.29)	120 (29.64)	
≥30	608 (41.98)	465 (42.27)	143 (40.87)	
Smoke status, *n* (%)				<0.001
Former	242 (17.52)	208 (19.38)	34 (10.32)	
Never	674 (44.96)	458 (42.48)	216 (54.59)	
Now	541 (37.52)	402 (38.14)	139 (35.09)	
Recreational activity, *n* (%)				<0.0001
No	1,035 (64.83)	725 (61.28)	310 (78.60)	
Yes	422 (35.17)	343 (38.72)	79 (21.40)	
DM, *n* (%)				<0.0001
No	824 (60.56)	647 (64.35)	177 (45.83)	
pre-DM	150 (10.80)	107 (10.08)	43 (13.57)	
Yes	483 (28.64)	314 (25.56)	169 (40.59)	
Hypertension, *n* (%)				<0.0001
No	494 (39.23)	394 (42.56)	100 (26.29)	
Yes	963 (60.77)	674 (57.44)	289 (73.71)	
CVD, *n* (%)				<0.0001
No	953 (69.59)	739 (73.03)	214 (56.27)	
Yes	504 (30.41)	329 (26.97)	175 (43.73)	
Cancer, *n* (%)				0.02
No	1,158 (76.37)	876 (77.99)	282 (70.07)	
Yes	299 (23.63)	192 (22.01)	107 (29.93)	
Alt (U/L)	25.00 (1.04)	24.49 (0.74)	26.98 (4.00)	0.54
Ast (U/L)	26.22 (0.65)	25.41 (0.66)	29.36 (1.98)	0.06
Creatinine (mg/dl)	0.94 (0.01)	0.92 (0.01)	1.03 (0.03)	0.001
Uric acid (mg/dl)	5.64 (0.05)	5.55 (0.06)	5.97 (0.13)	0.004
WBC (1000cells/ul)	7.75 (0.08)	7.71 (0.09)	7.88 (0.18)	0.39
Time (months)	76.80 (2.18)	81.96 (2.51)	56.80 (3.04)	<0.0001
DII	1.70 (0.08)	1.60 (0.08)	2.10 (0.11)	<0.0001
CDAI	0.16 (0.15)	0.37 (0.16)	−0.65 (0.19)	<0.0001

n, numbers; PIR, poverty income ratio; DM, diabetes mellitus; BMI, body mass index; CVD, Cardiovascular disease; ALT, alanine transaminase; AST, aspartate transaminase; WBC, white blood cells; DII, dietary inflammatory index; CDAI, composite dietary antioxidant index.

**Table 2:** Univariate analysis between variables and survival state, weighted.

Variables	HR(95% CI)	*p*- value
Age (years)		
<60	ref	ref
≥60	4.44 (3.13,6.28)	<0.0001
Gender		
Female	ref	ref
Male	0.87(0.68,1.11)	0.27
Race		
Non-Hispanic White	ref	ref
Non-Hispanic Black	1.01 (0.77,1.33)	0.95
Mexican American	0.57 (0.34,0.94)	0.03
Other Hispanic	0.68 (0.31,1.49)	0.34
Other race	0.62 (0.31,1.24)	0.18
Education level		
Less than high school	ref	ref
High School or equivalent	0.71 (0.52,0.98)	0.04
College Graduate or above	0.61 (0.45,0.83)	0.002
PIR		
<1	ref	ref
1–3	0.92 (0.70,1.20)	0.52
≥3	0.39 (0.27,0.55)	<0.0001
BMI		
<25	ref	ref
25–30	0.74 (0.52,1.04)	0.08
≥30	0.97 (0.74,1.27)	0.83
Smoke status		
Former	ref	ref
Never	2.31 (1.52,3.50)	<0.0001
Now	1.73 (1.09,2.75)	0.02
Recreational activity		
No	ref	ref
Yes	0.42 (0.29,0.62)	<0.0001
DM		
No	ref	ref
pre-DM	1.89 (1.23,2.92)	0.004
Yes	2.68 (1.96,3.67)	<0.0001
Hypertension		
No	ref	ref
Yes	2.12 (1.61,2.79)	<0.0001
CVD		
No	ref	ref
Yes	2.56 (2.07,3.17)	<0.0001
Cancer		
No	ref	ref
Yes	1.66 (1.23,2.25)	<0.001
Alt	1.00 (1.00,1.01)	0.37
Ast	1.01 (1.00,1.01)	<0.001
Creatinine	1.60 (1.39,1.83)	<0.0001
Uric acid	1.16 (1.04,1.28)	0.01
WBC	1.06 (1.00,1.13)	0.04
Time	0.00 (0.00,0.00)	<0.0001
DII	1.16 (1.10,1.22)	<0.0001
CDAI	0.93 (0.90,0.96)	<0.0001

PIR, poverty income ratio; DM, diabetes mellitus; BMI, body mass index; CVD, Cardiovascular disease; ALT, alanine transaminase; AST, aspartate transaminase; WBC, white blood cells; DII, dietary inflammatory index; CDAI, composite dietary antioxidant index.

Adjusting for all potential confounding factors, weighted multivariable Cox regression analysis revealed that both the crude model and the adjusted model indicated a positive association between DII and all-cause mortality in COPD, while CDAI was negatively associated with all-cause mortality (Table 3).

**Table 3:** Multivariate cox regression analysis of the correlation between DII or CDAI and all-cause mortality, weighted.

Variables	Crude model		Model 1		Model 2	
	HR (95% CI)	*p*	HR (95% CI)	*p*	HR (95% CI)	*p*
DII						
Continuous variables	1.16 (1.10,1.22)	<0.0001	1.19 (1.12,1.26)	<0.0001	1.11 (1.04,1.18)	0.002
Categorical variables						
Q1	ref		ref		ref	
Q2	1.80 (1.22,2.67)	0.003	1.90 (1.27,2.82)	0.002	1.71 (1.18,2.48)	0.004
Q3	1.78 (1.33,2.38)	<0.001	1.97 (1.47,2.63)	<0.0001	1.36 (1.00,1.85)	0.05
*p* for trend		<0.0001		<0.0001		0.02
CDAI						
Continuous variables	0.93 (0.90,0.96)	<0.0001	0.93 (0.89,0.96)	<0.0001	0.95 (0.91,0.99)	0.01
Categorical variables						
Q1	ref		ref		ref	
Q2	1.11 (0.82,1.50)	0.50	1.13 (0.82,1.55)	0.44	1.30 (0.97,1.75)	0.08
Q3	0.52 (0.37,0.74)	<0.001	0.50 (0.35,0.71)	<0.0001	0.63 (0.43,0.92)	0.02
*p* for trend		<0.0001		<0.0001		0.01

Crudel model: Covariates were not adjusted. Model 1: Adjusted by age, gender, race. Model 2: Adjusted by age, gender, race, education level, poverty income ratio, body mass index, smoking history, white blood cells, aspartate transaminase, creatinine, urea nitrogen, exercise, hypertension, diabetes mellitus, cardiovascular disease, and cancer history. HR, hazard ratios; CI, Confidence Interval; DII, dietary inflammatory index; CDAI, composite dietary antioxidant index. Q1-3, respectively, represent the groups divided according to the tertiles of DII: Q1 (≤1.18 mg/day), Q2 (1.18 to 2.98 mg/day), Q3 (>2.98 mg/day); and the tertiles of CDAI: Q1 (≤ −2.15 mg/day), Q2 (−2.15 to 0.69 mg/day), Q3 (>0.69 mg/day).

Compared to the lowest tertile of DII, the weighted multivariate HR (95% CI) of all-cause death was 1.71(1.18, 2.48) in Q2 and 1.36 (1.00, 1.85) in Q3 (*P* for trend = 0.02). In addition, using the Q1 level of CDAI as a reference, the HR (95% CI) of Q2 and Q3 were 1.30 (0.97, 1.75), 0.63 (0.43, 0.92) respectively (*p* = 0.01) (Table 3).

**Figure 2:**
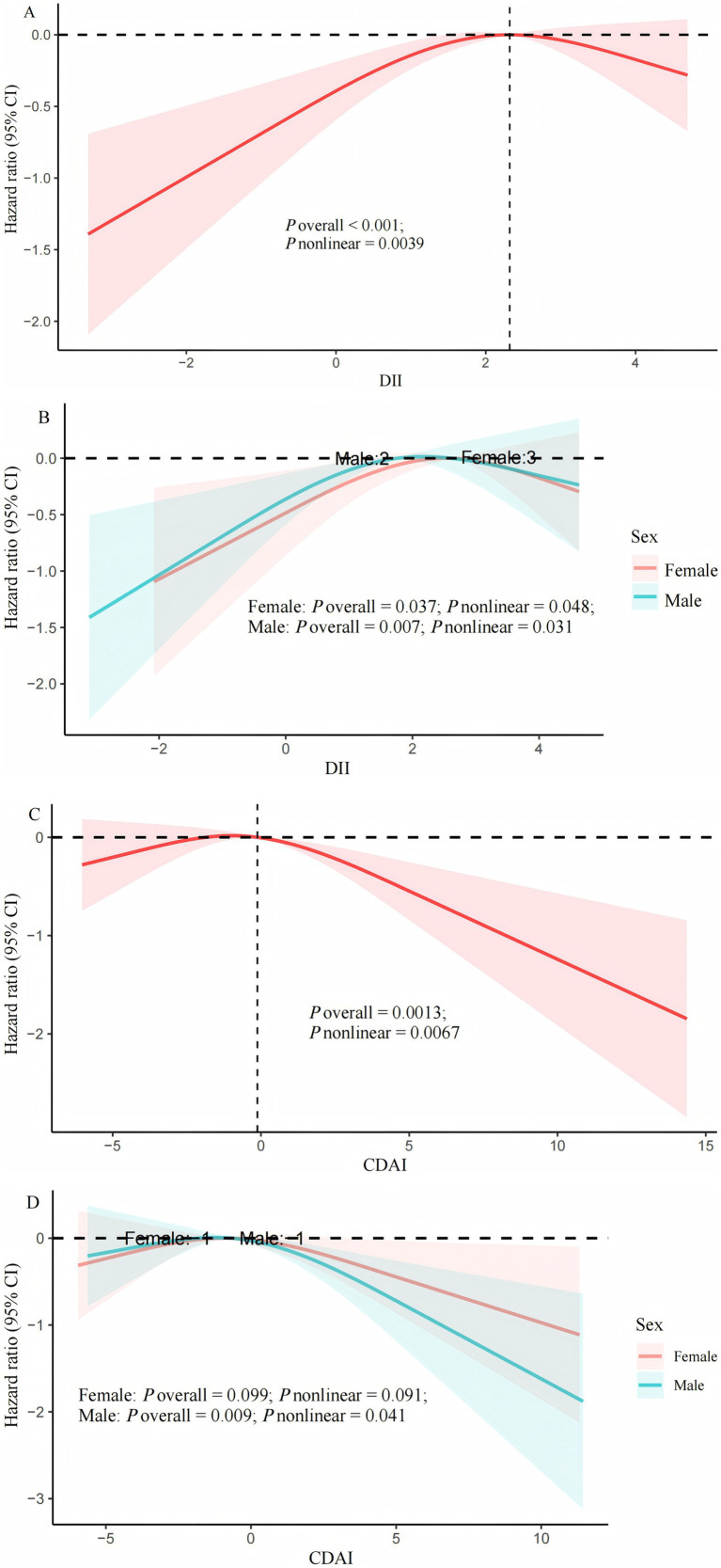
Cox regression using restricted cubic spline regression of DII and CDAI with all-cause mortality. **(A)** multivariable Cox regression between DII and all-cause mortality; **(B)** multivariable Cox regression between DII and all-cause mortality stratified by sex; **(C)** multivariable Cox regression between CDAI and all-cause mortality; **(D)** multivariable Cox regression between CDAI and all-cause mortality stratified by sex. Adjusted by age, gender, race, education level, poverty income ratio, body mass index, smoking history, white blood cells, aspartate transaminase, creatinine, urea nitrogen, exercise, hypertension, diabetes mellitus, cardiovascular disease, and cancer history. DII, dietary inflammatory index; CDAI, composite dietary antioxidant index.

1. After adjusting for all potential covariates, RCS showed a nonlinear correlation between DII or CDAI and all-cause death (*P*-nonlinear <0.05, Figure 2A; Figure 2C). When stratified by gender, these nonlinear correlations still existed among male and female COPD patients (Figure 2B; Figure 2D).
2. Threshold effect analysis indicated that the threshold value of DII was 2.32. Below 2.32, for every unit increase in DII, the risk of all-cause death increased by 23% [HR (95% CI): 1.23 (1.05, 1.44); *p* = 0.01]. Above 2.32, no association between the two was observed [HR 0.86; 95% CI (0.63, 1.18); *p* = 0.36; Table 4].

**Table 4:** Threshold effect analysis of DII and CDAI with all-cause mortality using piece-wise Cox regression.

Variable (DII)	HR (95% CI)	*p* value
Model I		
One line effect	1.06 (1.00, 1.13)	0.035
Model II		
Infection point (K)	2.32	
DII < 2.32	1.17 (1.07, 1.29)	0.001
DII > 2.32	0.86 (0.73, 1.02)	0.077
*p* for log-likelihood ratio test		0.007
Variable (CDAI)		
Model I		
One line effect	0.97 (0.94, 1.00)	0.041
Model II		
Infection point (K)	−0.12	
CDAI < −0.12	1.07 (0.99, 1.15)	0.075
CDAI > −0.12	0.91 (0.87, 0.96)	0.001
*p* for log-likelihood ratio test		0.003

Model I, one-line linear regression; Model II, two-segment regression. HR was the effect size and the 95% CI indicated the confidence interval. Adjusting for age, gender, race, education level, poverty income ratio, body mass index, smoking history, white blood cells, aspartate transaminase, creatinine, urea nitrogen, exercise, hypertension, diabetes mellitus, cardiovascular disease, and cancer history. DII, dietary inflammatory index; CDAI, composite dietary antioxidant index.

The threshold for CDAI associated with all-cause mortality is −0.12. When CDAI > −0.12, for every unit increase in CDAI, the risk of all-cause mortality decreased by 9% [HR (95% CI): 0.91 (0.87, 0.96); *p* = 0.001]; When CDAI < −0.12, it was not associated with all-cause mortality [HR (95% CI): 1.07 (0.99, 1.15); *p* = 0.075].

**Figure 3:**
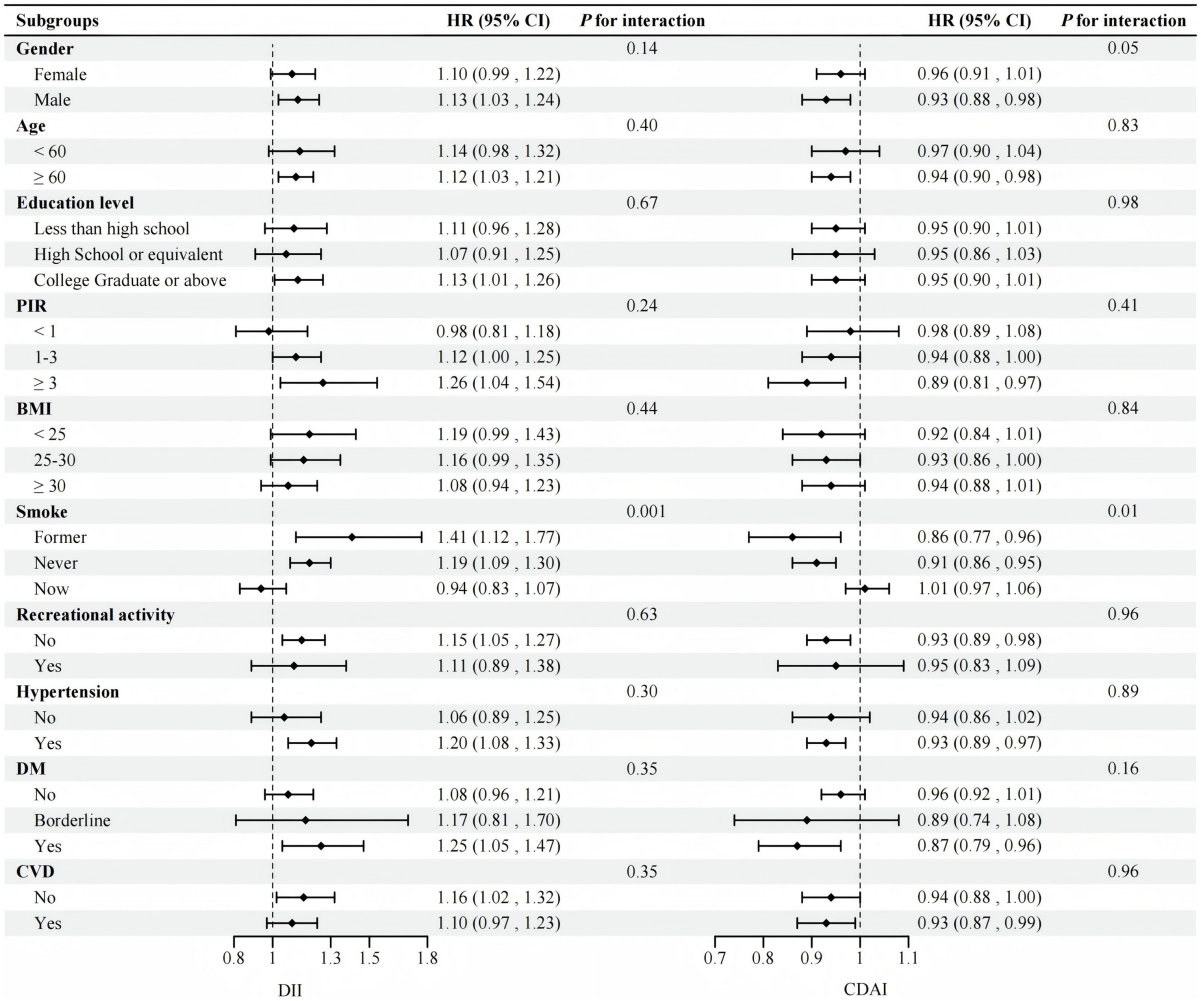
Stratified analyses between DII and CDAI and all-cause mortality (*N* = 1,457). Adjusted by age, gender, race, education level, PIR, BMI, smoking history, white blood cells, aspartate transaminase, creatinine, urea nitrogen, exercise, hypertension, DM, CVD, and cancer history except for the stratification variable. PIR, poverty income ratio; BMI, body mass index; DM, diabetes mellitus; CVD, Cardiovascular disease; DII, dietary inflammatory index; CDAI, composite dietary antioxidant index.

1. Stratified analysis and interaction tests were carried out in terms of gender, age, education, PIR, BMI, smoking, exercise, and comorbidities (hypertension, diabetes, and CVD). The results continued to confirm that the impact of DII or CDAI on all-cause mortality in most subgroups was similar to that in the general population (*P* for interaction >0.05). The impact of exposed variables on all-cause mortality was more pronounced in the never-smoked or past-smoker subgroups (*P* for interaction <0.05) (Figure 3).

## Discussion

To our knowledge, this survey was the first to explore the impact of DII and CDAI on all-cause mortality in COPD patients. After adjusting for potential confounding factors, there was a nonlinear association between DII or CDAI and all-cause deaths, with inflection points of 2.32 and − 0.12, respectively. The results showed that higher DII and lower CDAI increased all-cause deaths from COPD. In addition, these relationships were consistent across many subgroups. Pro-inflammatory diets or antioxidant diets have a more pronounced effect on death in non-smokers.

Chronic airway inflammation is one of the main characteristics of COPD. Airway inflammation is associated with the progression and mortality of COPD disease (22, 23). Data from Pan MH et al. paper stated that dietary factors can alter the expression of inflammatory genes in the human body (24). Epidemiological studies have found that a higher DII score was positively correlated with the risk of COPD or early COPD and a decline in lung function (25, 26). We further analyzed the impact of pro-inflammatory diets on all-cause mortality in patients with COPD, which was consistent with the research of Tian et al. (17). These findings support that dietary modification can be an intervention to improve outcomes in patients with COPD.

The mechanism by which DII affects all-cause mortality in COPD patients might involve elevating inflammatory factors. A large number of studies have confirmed that DII is positively associated with inflammatory markers (e.g., CRP, IL-6, TNF-*α*, IL-1, 2, IFN-*γ*) of a variety of chronic diseases (27–32). Consumption of vegetables, fruits, and legumes rich in fiber and vitamins is associated with lower markers of inflammation (33–35). This suggests that dietary factors may affect the prognosis of COPD by affecting the level of inflammatory markers in the body. It is suggested that anti-inflammatory diets may help reduce inflammatory responses in the body. We should pay attention to the impact of unhealthy eating habits on public health.

Oxidative stress is the primary driving mechanism in the development of COPD (36, 37). Smoke, air pollution, and lung inflammation generate reactive oxygen species, leading to an increase in oxidative stress in COPD patients. This oxidative stress further amplifies chronic inflammation and accelerates lung aging (36). Many studies have shown that an antioxidant-rich diet can lower the risk of COPD, improve lung function, and improve inflammatory status (38–43). Two cross-sectional studies have revealed that CDAI can reduce the risk of chronic respiratory diseases and COPD (18, 44). A cross-sectional study from the United States has indicated that CDAI is positively correlated with FEV1, FVC, and PRISm (19). However, previous research has not investigated the impact of CDAI on all-cause mortality in COPD. This study found that CDAI can reduce all-cause mortality in COPD, with a turning point at −0.12. When CDAI > −0.12, with an increase in CDAI, the all-cause mortality in COPD patients decreased. Moreover, this negative correlation still existed across most subgroups. This indicates that increasing antioxidant-rich diets may be an effective strategy to improve the prognosis of COPD.

The pro-inflammatory diet mainly includes alcohol, processed meat, red meat (such as pigs, cattle, and lamb), refined carbohydrates (such as cakes), fried foods, sugary beverages, and saturated fatty acids (45). Vegetables, fruits, dietary fiber, legumes, whole grains, tea, coffee, and foods rich in unsaturated fatty acids (such as nuts, fish, melon seeds, and olives) are called anti-inflammatory foods (46). Diet has the potential to regulate inflammation and influence the outcomes of infections and chronic diseases (47). The dietary pyramid of patients with COPD pointed out that it is beneficial to adjust their lifestyle by increasing the intake of a large number of fruits and vegetables, dietary fiber, and antioxidants to improve FEV1 and reduce oxidative stress (48). It is recommended that patients with COPD should reduce the intake of pro-inflammatory foods and increase the intake of foods with specific antioxidant functions.

This study has some advantages and disadvantages. It is the first to analyze the impact of exposure factors DII and CDAI on all-cause mortality in COPD, using complex sampling weighting analysis to represent the general COPD patients in the United States, and adjusting for as many potential confounding effects as possible. Of course, the research has its limitations. First of all, from 2013 to 2018, the lack of pulmonary function data in the database led to the diagnosis of COPD relying on questionnaires, which may have resulted in either overestimation or underestimation. This method does not fully reflect the true situation of COPD. If this part of the population has spirometry data, it will improve the robustness of our research results. Secondly, dietary recall data cannot represent long-term dietary habits, which limits our analysis of the relationship between changing dietary factors and all-cause death. In addition to further strengthening large-scale prospective studies in the future, intervention trial studies should also be gradually carried out to provide stronger evidence for clarifying the relationship of DII and CDAI with COPD.

Adopting a healthier diet as part of a healthy lifestyle to protect lung function and prevent or improve COPD has great potential to improve public health (49). In addition to encouraging avoidance or quitting smoking, our results revealed a maximum pro-inflammatory inflection point of the diet is 2.32, while the ideal CDAI level of the antioxidant diet is above −0.12. We recommend eating more antioxidant foods and less inflammatory foods under the premise of balanced nutrition, following current nutritional guidelines such as the COPD diet pyramid.

## Conclusion

These findings indicate that inflammatory diets can increase the all-cause mortality risk in COPD patients, while antioxidant diets can decrease all-cause mortality in COPD. It suggests that healthy dietary patterns can be used as interventions and management measures for COPD. Of course, further confirmation of the relationship between the two would require additional cohort studies and research into potential mechanisms.

## Data Availability

Publicly available datasets were analyzed in this study. This data can be found at: https://www.cdc.gov/nchs/nhanes/.
